# A new sauropod dinosaur hindlimb from the Lower Cretaceous Wessex Formation, Isle of Wight, UK

**DOI:** 10.1098/rsos.240642

**Published:** 2024-10-30

**Authors:** Robert R. Higgins, Philip D. Mannion, Paul M. Barrett, Paul Upchurch

**Affiliations:** ^1^ Department of Organismic and Evolutionary Biology, Harvard University, 26 Oxford Street, Cambridge, MA 02138, USA; ^2^ Department of Earth Sciences, University College London, Gower Street, London WC1E 6BT, UK; ^3^ Fossil Reptiles, Amphibians and Birds Section, Natural History Museum, Cromwell Road, London SW7 5BD, UK

**Keywords:** Cretaceous, Flagellicaudata, Sauropoda, Somphospondyli, Titanosauriformes

## Abstract

The Barremian-aged Wessex Formation of the Isle of Wight, UK, offers a globally significant glimpse into the sauropod dinosaur faunas of the early Cretaceous. These deposits have yielded specimens of several neosauropod lineages, such as rebbachisaurids, titanosauriforms (including some of the earliest titanosaur remains), and possible flagellicaudatans. Here, we report an undescribed sauropod partial hindlimb from the Wessex Formation (NHMUK PV R16500) and analyse its phylogenetic affinities. This hindlimb preserves the left tibia, astragalus and pes, lacking only a few phalanges. NHMUK PV R16500 can be diagnosed based on two autapomorphies: an unusually high distal end to midshaft transverse width ratio in metatarsals III and IV, and the presence of small bump-like projections located in the centre of the proximal articular surfaces of the unguals of pedal digits I and II. The phylogenetic affinities of NHMUK PV R16500 are uncertain: although our analyses recover it as an early-branching somphospondylan, a single character change moves it to close to Flagellicaudata when extended implied weighting is applied. The possibility of flagellicaudatan affinities for NHMUK PV R16500 implies a potential ghost lineage that survived the Jurassic/Cretaceous boundary; however, we present evidence that the somphospondylan position is more probable and should be preferred.

## 1. Introduction

Since the first discoveries in the mid-nineteenth century, the formations of the Lower Cretaceous Wealden Supergroup of southern England have yielded numerous sauropod dinosaur remains [1–10]. However, most of these specimens are fragmentary and do not overlap anatomically, leading to taxonomic and nomenclatural confusion [10]. During the past two decades, several authors have re-described and/or reviewed elements of these Early Cretaceous sauropod faunas, resulting in a clearer picture of their contents [7–18]. In particular, the Barremian-aged beds of the Wessex Formation on the Isle of Wight have yielded a diverse sauropod fauna composed of rebbachisaurid diplodocoids, early-branching somphospondylans and putative brachiosaurids, some of the earliest known titanosaurian remains, and more enigmatic specimens such as the tooth of *Oplosaurus* that might represent a late-surviving non-neosauropod eusauropod lineage [10,13,16,19–22].

Despite this progress, which has been facilitated by an improved understanding of sauropod phylogeny, the fragmentary nature of the available specimens and scarcity of material with overlapping anatomy continues to hinder the establishment of a more complete, accurate picture of the Isle of Wight sauropod fauna, limiting the ability to make comparisons with contemporaneous taxa from mainland Europe and elsewhere. Articulated or associated sauropod specimens from the Wealden Supergroup are rare and therefore of exceptional interest. Here, we describe an associated left tibia, astragalus and almost complete pes of a sauropod dinosaur from the Wessex Formation of the Isle of Wight. We make comparisons with a broad array of eusauropods, including contemporaneous western Eurasian taxa, and incorporate it into a modified phylogenetic dataset to establish its affinities.

## 2. Abbreviations

Institutions: NHMUK, The Natural History Museum, London, UK; MAU, Museo Argentino Urquiza, Rincón de los Sauces, Neuquén, Argentina; CAMSM, Sedgwick Museum, University of Cambridge, Cambridge, UK.

## 3. Systematic palaeontology

Dinosauria [23]

Sauropoda [24]

Neosauropoda [25]

Incertae sedis

Gen. et sp. indet.

### 3.1. Specimen

**Figure 1.**
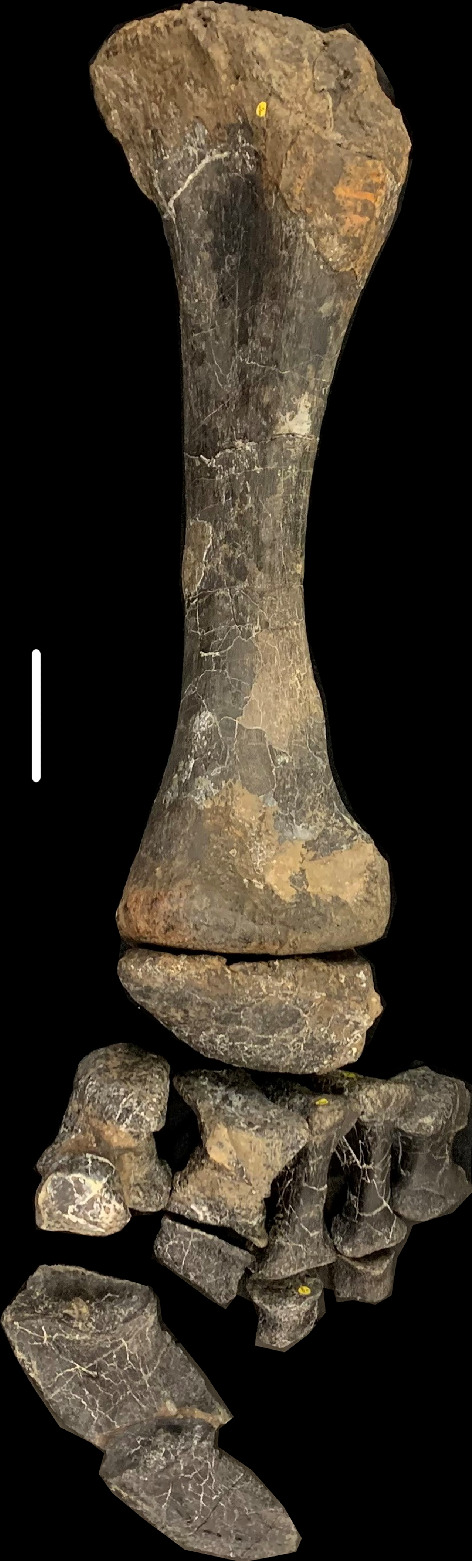
Anterior view of left hindlimb NHMUK PV R16500, in a semi-articulated position, comprising: a left tibia, left astragalus, left metatarsals I–V and left phalanges I-1, II-1, III-1, IV-1, I-2 and II-3. Scale bar = 100 mm.

NHMUK PV R16500 (figure 1): a left tibia, astragalus, metatarsals I–V, pedal phalanges I-1, II-1, III-1, IV-1 and the unguals of digits I and II, found in association. Note: two elements, originally identified as caudal vertebrae, were also found close to this specimen and were initially accessioned under the same NHMUK register number. However, closer examination indicates that these are the cervical centra of an ornithischian dinosaur and they have subsequently been assigned the specimen number NHMUK PV R38864 (these specimens will not be considered further here).

### 3.2. Locality and horizon

Sudmore Point, south of Brook, Isle of Wight, UK; Wessex Formation, lower Barremian, Lower Cretaceous. This specimen was collected by Alan Parfitt and donated to the NHMUK in 2002. Additional information in the accession notes indicates that the specimen was found in the ‘Sudmore Point sandstone member’, an informal division of the Wessex Formation, that outcrops between Brook Chine and Chilton Chine on the southwest coast of the Isle of Wight (see [26, pp. 87–90 and fig. 23]). This sandstone occurs just above the Hauterivian/Barremian boundary ([27]; see also [28]), and we therefore regard its stratigraphic age as early Barremian.

## 4. Description and comparisons

### 4.1. Left tibia

The left tibia is complete and has suffered relatively little distortion. The proximal articular surface is rugose and nearly flat, with a mild concavity on its medial portion and slight convexity towards its lateral edge. This articular surface curves distally as it merges into the proximal parts of the cnemial crest and lateral process for articulation with the fibula. In proximal view, this surface is subcircular in outline, with subequal transverse and anteroposterior diameters (table 1). A tibia with a subcircular proximal end was originally regarded as a neosauropod synapomorphy [29], but it is probably to characterize a more inclusive clade approximating to Eusauropoda, given that this condition also occurs in *Bagualia alba*, *Omeisaurus tianfuensis*, *Mamenchisaurus youngi*, *Mierasaurus bobyoungi*, *Janenschia robusta* and *Jobaria tiguidensis* [30]. Some turiasaurs [31] and neosauropods have a more transversely compressed and anteroposteriorly elongated proximal surface, including *Euhelopus zdanskyi* [32] and some rebbachisaurids [33,34]. In lateral view, the proximal articular surface of NHMUK PV R16500 is oriented perpendicular to the long axis of the tibial shaft.

**Table 1.** Measurements of the elements preserved in NHMUK PV R16500.

element	measurement type	measurement (mm)
tibia	length	684
	proximal end mediolateral width	215
	proximal end anteroposterior length	206
	midshaft maximum diameter	115
	midshaft minimum circumference	310
	distal end maximum mediolateral width	203
	distal end maximum anteroposterior length	161
astragalus	mediolateral width	213
	anteroposterior length	133
	proximodistal height	112
metatarsal I	length	115
	proximal end maximum dorsoventral height	106
	proximal end maximum mediolateral width	79
	midshaft mediolateral width	61
	distal end maximum dorsoventral height	66
	distal end maximum mediolateral width	109
metatarsal II	length	134
	proximal end maximum dorsoventral height	117
	proximal end maximum mediolateral width	74
	midshaft mediolateral width	51
	distal end maximum dorsoventral height	58
	distal end maximum mediolateral width	89
metatarsal III	length	152
	proximal end maximum dorsoventral height	102
	proximal end maximum mediolateral width	53
	midshaft mediolateral width	24
	distal end maximum dorsoventral height	55
	distal end maximum mediolateral width	75
metatarsal IV	length	144
	proximal end maximum dorsoventral height	84
	proximal end maximum mediolateral width	58
	midshaft mediolateral width	24
	distal end maximum dorsoventral height	50
	distal end maximum mediolateral width	69
metatarsal V	length	120
	proximal end maximum dorsoventral height	61
	proximal end maximum mediolateral width	102
	midshaft mediolateral width	47
	distal end maximum dorsoventral height	31
	distal end maximum mediolateral width	74
phalanx I-1	length	47
	proximal end width	69
	proximal end dorsoventral height	69
phalanx II-1	length	48
	proximal end width	77
	proximal end dorsoventral height	65
	distal end width	72
phalanx III-1	length	48
	proximal end width	62
	proximal end dorsoventral height	56
phalanx IV-1	length	42
	proximal end width	58
	proximal end dorsoventral height	42
ungual I	length	190 (distal tip missing)
	proximal end dorsoventral height	112
	proximal end mediolateral width	64
ungual II	length	142
	proximal end dorsoventral height	89
	proximal end mediolateral width	50

**Figure 2.**
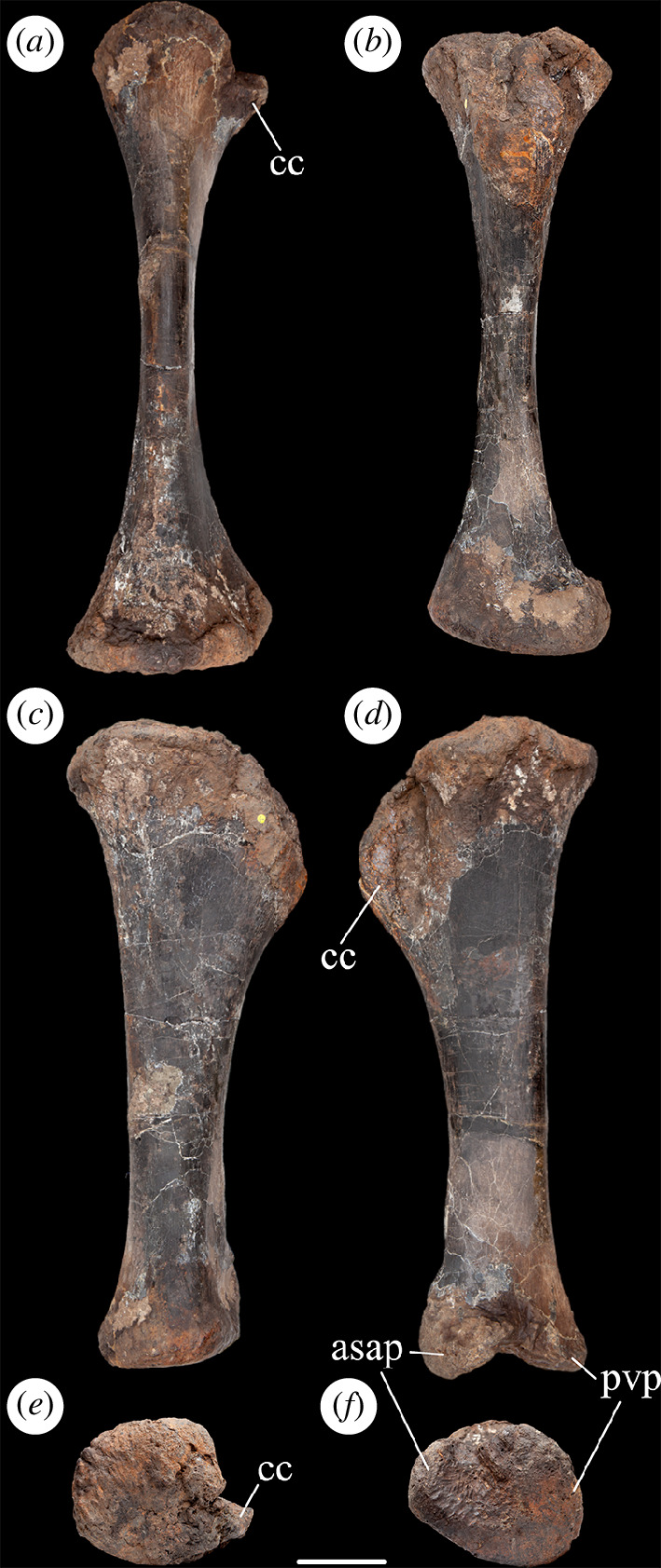
Left tibia of NHMUK PV R16500, in: (*a*) anterior; (*b*) posterior; (*c*) medial; (*d*) lateral; (*e*) proximal; and (*f*) distal views. Scale bar = 100 mm; asap, articular surface of the ascending process; cc, cnemial crest; pvp, posteroventral process.

As is typical for many eusauropods [29], including the majority of diplodocoids [14,35] and the contemporaneous European somphospondylan *Garumbatitan morellensis* [36], the cnemial crest projects primarily laterally rather than anteriorly. This orientation is achieved owing to the cnemial crest curving laterally from an anterolaterally projecting base. In this regard, the tibia differs from that of the contemporaneous European somphospondylan *Tastavinsaurus sanzi*, as well as the rebbachisaurid *Nigersaurus taqueti*, which are each characterized by an anterolaterally deflected cnemial crest [16,37]. There are no discernible ridges or scars on either the medial or lateral surfaces of the cnemial crest, but these regions are slightly damaged and eroded. In anterior view, the free (i.e. lateral) margin of the cnemial crest is convex, with its most prominent point located well below the level of the proximal articular end (figure 2*a*). There is no evidence of a ‘tuberculum fibularis’ on the posterior surface of the cnemial crest, contrasting with the condition in some brachiosaurids and flagellicaudatans, as well as a small number of non-neosauropods [35,38]. Posterior to the cnemial crest, and anterior to the lateral process, there is a deep vertical groove that probably received the anterior part of the proximal end of the fibula. Unlike the tibiae of many sauropods, including *Garumbatitan* and *Tastavinsaurus* [36,37], a ’second cnemial crest’ is absent in NHMUK PV R16500, as is also the case in a range of distantly related taxa such as *Jobaria*, *Bellusaurus sui*, *Tehuelchesaurus benitezii*, *Sauroposeidon proteles*, *Euhelopus* and *Malawisaurus dixeyi* [16]. The lateral process is broadly convex in proximal view (figure 2*e*). This process fades out distally after a short distance as it merges into the lateral face of the shaft.

The minimum diameter of the tibial shaft occurs at midlength. At this point, the shaft has a rounded, ‘D’-shaped cross-section, formed from a flattened lateral surface and rounded medial one. The shaft is relatively constricted compared with the proximal and distal ends of the tibia, such that the transverse width of the distal end is approximately 1.8 times the maximum diameter of the shaft at midlength (table 1). Values of this ratio below 2.0 are typical for most sauropods, apart from a few titanosaurs such as *Malawisaurus*, *Opisthocoelicaudia skarzynskii* and *Saltasaurus loricatus*, where it exceeds 2.0 [14,16,39]. On the medial surface, near the proximal end of the shaft, there is a vertical ridge located close to the posterior margin. This separates a smaller vertical fossa posteriorly from a larger shallow concavity anteriorly. However, there is some cracking in this region and we suspect that this ridge and associated concavities are probably the result of crushing. Towards its distal end, the shaft develops a subtriangular cross-section because of the transverse widening of the anterior face. This creates a shallowly concave subtriangular area on the anterior surface of the tibia at its distal end, as occurs in most sauropods [40].

The distal articular surface is flat and expanded both transversely and anteroposteriorly. There is no ridge or raised area on the medial surface of the distal end. The robustness index is 0.26, and the ratio of the transverse to anteroposterior diameters of the distal end is 1.3: similar values close to 1.0 occur in most eusauropods, except for several titanosaurs (e.g. *Alamosaurus sanjuanensis*, *Opisthocoelicaudia* and *Saltasaurus*) and a few other taxa such as *Chuanjiesaurus anaensis* and *Mamenchisaurus youngi*, where the ratio is typically greater than 1.5 [14,16,41,42]. As in other eusauropods [29], the medial malleolus is reduced in transverse width: this results in the exposure of the ascending process of the astragalus in posterior view when these two elements are articulated. The lateral malleolus extends further laterally than the medial one, but the latter projects slightly further distally than the former. On the posterolateral surface of the distal end, the two malleoli are separated by a shallow vertical groove. In distal end view, the articular surface for the astragalus has a subtriangular or heart-shaped outline.

### 4.2. Left astragalus

**Figure 3.**
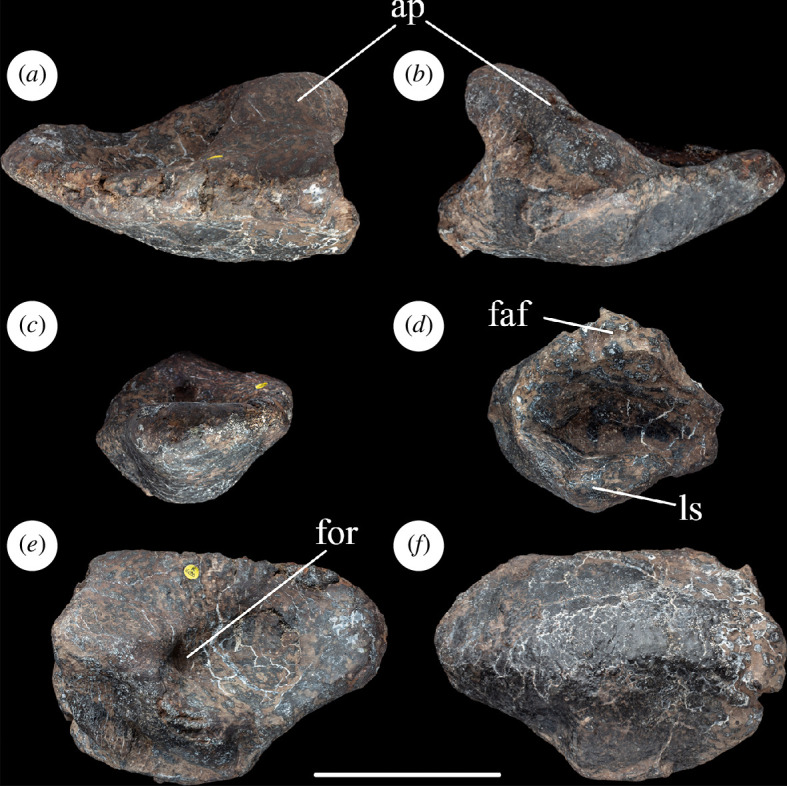
Left astragalus of NHMUK PV R16500, in: (*a*) anterior; (*b*) posterior; (*c*) medial; (*d*) lateral; (*e*) proximal; and (*f*) distal views. Scale bar = 100 mm; ap, ascending process; faf, fibular articular facet; for, foramen; ls, lateral shelf.

The astragalus is complete and nearly undistorted. In anterior and proximal views (figure 3*a,e*), the astragalus tapers towards its medial end: this is the apomorphic condition that occurs in neosauropods and some closely related eusauropods [43]. When articulated with the tibia, the astragalus completely caps the distal end of the latter element, as in most sauropodomorphs, contrasting with the apomorphic reduction of the astragalus and related exposure of part of the distal end of the tibia seen in most titanosauriforms and rebbachisaurids, with exceptions including *Dongbeititan dongi*, *Euhelopus*, *Garumbatitan* and *Vouivria damparisensis* [16,32,36,44,45]. The transverse width to proximodistal height ratio of the astragalus of NHMUK PV R16500 is 1.9: this is the plesiomorphic state that occurs in most other sauropods and contrasts with ratios below 1.8 present in several saltasaurids and sporadically among other taxa, such as *Nigersaurus*, *Dicraeosaurus hansemanni*, *Tornieria africana* and *Chuanjiesaurus* [14,16,39]. In NHMUK PV R16500, the ratio of the transverse width of the astragalus to its anteroposterior width at its lateral end is 1.6: again, this is the plesiomorphic state and contrasts with ratios below 1.5 seen in various titanosauriforms and some diplodocoids [16,35,46].

As is also the case in other eusauropods [29], there is no pit or vascular foramen on the anterior surface of the astragalus at the base of the ascending process. The lateral surface of the ascending process forms an articular area for the distal end of the fibula. This surface faces mainly laterally and slightly proximally, and a moderate outward projection of its anterior margin means that this surface also faces slightly posteriorly. The deflection of the fibular articulation to face slightly proximally appears to be the result of taphonomic compression. Thus, the fibular articular surface of NHMUK PV R16500 lacks the laterally projecting shelf that is plesiomorphic for eusauropods and retained in some diplodocoids [16,32,35]. By contrast, the projection of the anterior margin of this articulation, such that it also faces mildly posteriorly, is potentially a synapomorphic state in NHMUK PV R16500 that, to date, has only been observed in diplodocoids [35,47] and the probable mamenchisaurid *Bellusaurus* [48,49].

The ascending process has a smooth proximal surface that slopes mildly distally towards its medial margin where it merges into the medial fossa. As in neosauropods [29], and some non-neosauropod eusauropods such as *Janenschia* and *Mierasaurus* [14,50], the ascending process extends to the posterior margin of the element. The posterior surface of this process bears a subtle, proximodistally oriented groove which is bounded laterally by the junction with the fibular articular surface and medially by a ridge that connects the posteromedial corner of the ascending process to the posterior margin of the astragalus. This medial ridge is plesiomorphically absent in non-sauropod sauropodomorphs and some early-diverging sauropods, such as *Spinophorosaurus nigerensis* [51], but is present in most sauropods apart from titanosaurs [29,52] and *Garumbatitan* [36]. As in many other sauropod astragali, NHMUK PV R16500 possesses a vascular foramen on the proximal surface, located just medial to the posterior end of the ascending process (but this is filled with matrix).

Most sauropod astragali (e.g. *Turiasaurus riodevensis*, *Vouivria* and *Oceanotitan dantasi*) possess a rounded tongue-like process that projects from the posterior margin, located just posteromedial to the ascending process [38,46,53]. However, in NHMUK PV R16500, this process is small and the groove separating it from the ascending process appears to be absent. The reduced nature of this feature could be regarded as the apomorphic loss that characterizes the astragali of many macronarians and *Dicraeosaurus* [14,16,46]: however, this process might have been folded downwards by crushing in NHMUK PV R16500, so we conservatively regard the presence or absence of this feature as equivocal and score it as a ‘?’ in our data matrix. The distal articular surface of the astragalus is strongly convex both anteroposteriorly and transversely, as occurs in most neosauropods and closely related eusauropods [43,54]. It is not possible to determine if the absence of a calcaneum is a genuine feature or reflects a preservational artefact (figure 3, table 1)

### 4.3. Pes

#### 4.3.1. Left metatarsal I

This is a short, robust element that generally resembles the first metatarsal of most neosauropods. It is the shortest of the five metatarsals (table 1) and is thus shorter than metatarsal V. This relationship between the lengths of metatarsals I and V is typical for sauropods, although there are a few cases where the former element is longer than the latter (e.g. turiasaurs, *Camarasaurus lentus*, and the titanosaurs *Rapetosaurus krausei* and *Opisthocoelicaudia*) [16]. The proximal articular end is ‘D’-shaped in outline, with a strongly convex medial margin and mildly concave lateral one. The articular surface itself is nearly flat transversely but more convex dorsoventrally, curving mildly distally as it approaches the dorsolateral and ventrolateral corners and dorsomedial margin. As a result, the proximal surface is bevelled with respect to the long axis of the shaft (facing proximally and moderately medially), as occurs in most neosauropods and several non-neosauropod eusauropods such as *Shunosaurus lii*, *Janenschia* and *Jobaria* [39]. The surface areas of the proximal ends of metatarsals I and V are similar to those of metatarsals II–IV (table 1), as is typical for sauropods [39], including early-diverging forms such as *Antetonitrus ingenipes* [55].

The ‘D’-shaped proximal outline persists into the transverse cross-section of the proximal part of the shaft, with the concave lateral margin leading to a subtriangular fossa that occupies the proximal half of the lateral surface. This fossa, for articulation with metatarsal II, is separated from the dorsal surface of the shaft by a ridge that extends distoventrally from the dorsolateral corner of the proximal end to the distolateral condyle. The ventral surface of the metatarsal is mildly convex transversely and strongly concave proximodistally owing to the marked expansion of the proximal end and more moderate expansion of the distal end. The medial surface of the shaft is narrow and mildly convex dorsoventrally and is separated from the dorsal and ventral surfaces by subtle longitudinal ridges, which has also been described in the rebbachisaurids *Agustinia ligabuei* and *Lavocatisaurus agrioensis* [33,34]. This medial surface lacks the bump-like projection at midlength observed in several brachiosaurids [14,56] and *Garumbatitan* [36]. The distal and medial part of the dorsal surface is obscured by the attached phalanx I-1. Unlike several diplodocids and the dicraeosaurid *Suuwassea emiliae* [54,57], NHMUK PV R16500 lacks a rugosity on the dorsolateral margin of the shaft, near the distal end. There is a ridge on the dorsolateral margin, but this appears to be a taphonomic alteration (there is damage immediately medial to this ridge) and, in any case, it occurs more proximally than the rugosity seen in flagellicaudatans [35].

**Figure 4.**
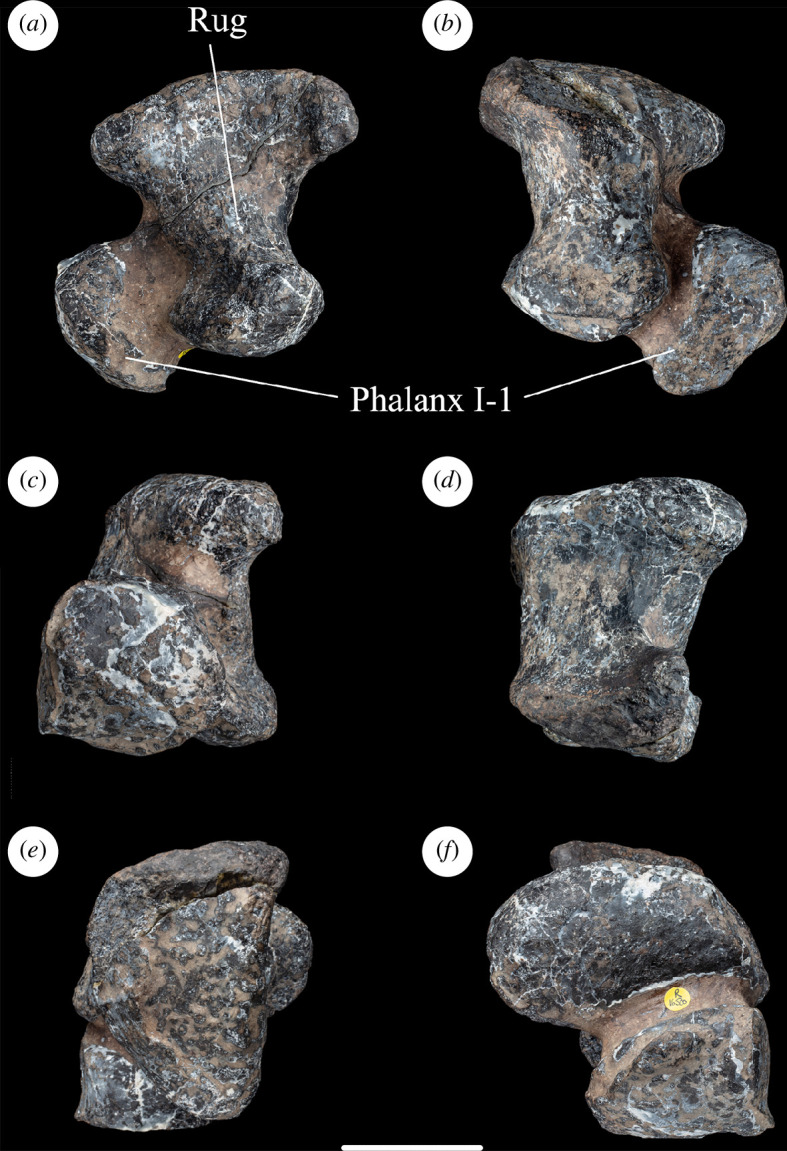
Left metatarsal I and phalanx I-1 of NHMUK PV R16500, in: (*a*) anterior; (*b*) posterior; (*c*) medial; (*d*) lateral; (*e*) proximal; and (*f*) distal views. Scale bar = 50 mm; Rug, rugosity.

The distal end of the metatarsal is strongly expanded transversely compared with the shaft, largely because of the expansion of the lateral margin towards the distal end. Thus, NHMUK PV R16500 possesses the apomorphic condition in which the distal end projects further laterally than the proximal end, as is also seen in several mamenchisaurids, flagellicaudatans and titanosauriforms [16,35,43], including the contemporaneous south-central Russian somphospondylan *Sibirotitan astrosacralis* [58], although this feature is absent in *Garumbatitan* and *Tastavinsaurus* [36,37]. The distal articular surface is nearly flat transversely and strongly convex dorsoventrally. Ventrally, the articulation extends only slightly onto the ventral surface. There is no midline groove dividing the distal articular surface to create a ginglymus. The lateral side of the metatarsal is longer proximodistally than the medial one, such that the distal end is mildly distomedially bevelled: this is the apomorphic condition that characterizes most sauropods [39,59]. There is no well-developed projection laterally from the ventral margin of the distolateral condyle in NHMUK PV R16500, such as that which occurs in many eusauropods [16,43], although there is a small, pointed process in this region. This projection, combined with mild excavation of the lateral surface of the distolateral condyle, means that this surface faces mildly dorsally and proximally as well as laterally. By contrast, the medial surface of the distomedial condyle is flat and faces moderately ventrally. In distal end view, the distomedial condyle is slightly wider dorsoventrally than the distolateral one (figure 4).

#### 4.3.2. Left metatarsal II

This is a robust element that resembles those of other sauropods in most respects. Application of the robustness measure (calculated as mean average proximal and distal transverse breadth divided by maximum length) used by Tschopp *et al.* [60] gives a value of 0.6 in NHMUK PV R16500: similar values are widespread among eusauropods, although some taxa (e.g. *Giraffatitan brancai*) possess a more gracile metatarsal II. The proximal end is more expanded dorsoventrally than transversely. In proximal end view, the articular surface is subrectangular, with a concave ventrolateral margin as seen in most sauropodomorphs, rather than the apomorphically straight margin that occurs sporadically among eusauropods (e.g. *Janenschia, Camarasaurus lentus, Diplodocus carnegii, Euhelopus*, *Ligabuesaurus leanzai* and *Saltasaurus* [16]). By contrast, the medial margin of the proximal end is very slightly concave to flat in NHMUK PV R16500. The proximal articulation is rugose and projects above the dorsal surface of the main shaft. This articular surface faces mainly proximally over most of its extent, but towards its dorsal margin, it extends mildly distally such that this region faces proximodorsally in lateral view. Near its lateral margin, this proximodorsal surface merges smoothly into the dorsal surface of the main shaft, whereas more medially the proximal and dorsal surfaces are separated from each other by a distinct transverse ridge. The ventral margin of the proximal articular surface is strongly convex transversely. This region is narrower transversely than the dorsal margin, largely because the latter projects laterally to overhang a fossa on the proximal half of the lateral surface of the shaft for articulation with metatarsal III.

**Figure 5.**
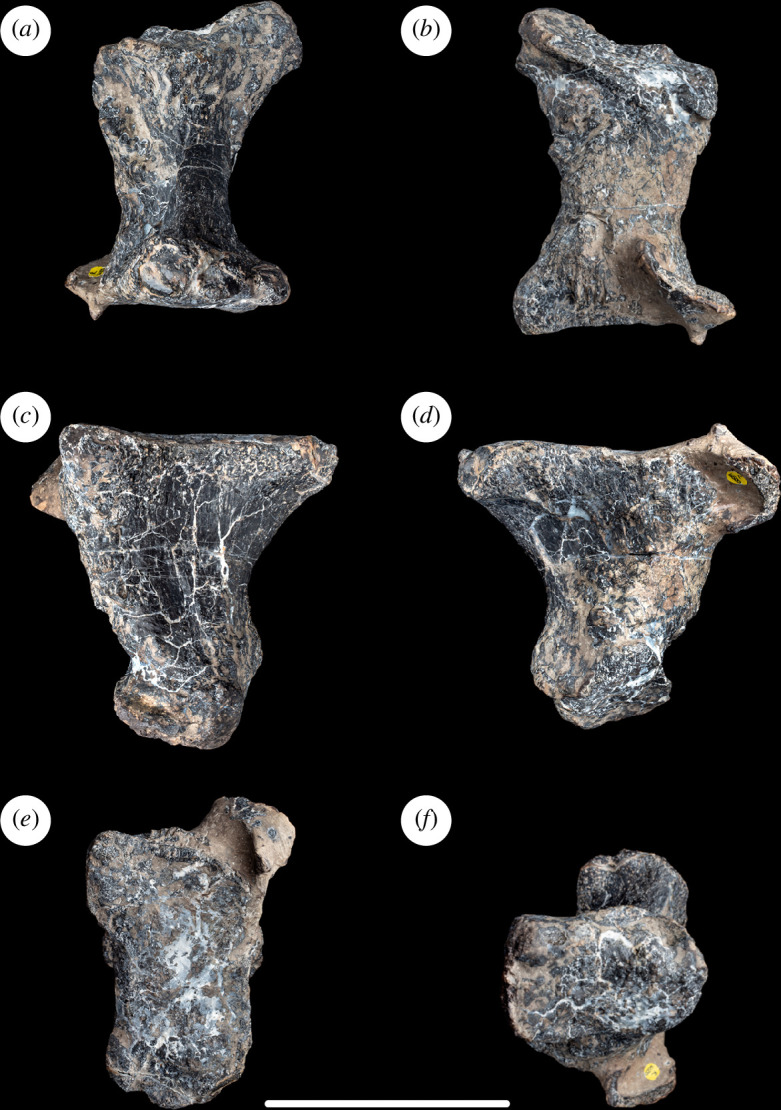
Left metatarsal II of NHMUK PV R16500, in: (*a*) anterior; (*b*) posterior; (*c*) medial; (*d*) lateral; (*e*) proximal; and (*f*) distal views. Scale bar = 100 mm.

The subrectangular profile of the proximal end persists into the shaft cross-section for a short way, but rapidly becomes more subtriangular, such that the dorsal surface is wider transversely than the rounded ventral surface. The dorsal surface of the shaft is too badly preserved to determine whether a dorsolateral rugosity was present near the distal end. Towards its distal end, the metatarsal widens strongly transversely, with most of this occurring in the form of a lateral expansion: thus, NHMUK PV R16500 possesses the apomorphic ventrolateral process at its distal end that occurs in many eusauropods, including *Tastavinsaurus*, although it is typically absent in most titanosauriforms [14,35]. The distal end is only very mildly expanded dorsoventrally. The distolateral condyle is formed by a transversely compressed and tall region that projects distinctly distolaterally: as a result, the metatarsal is longer on its lateral side than on its medial one, such that the distal end is set at a distinct angle relative to the long axis of the shaft in dorsal view. The subrectangular distal articular surface is convex dorsoventrally and curves up a short way onto the dorsal surface of the shaft, whereas it is moderately concave transversely (figure 5).

#### 4.3.3. Left metatarsal III

Metatarsal III is the longest metatarsal. This is the plesiomorphic state that occurs in most sauropods, contrasting with the apomorphically longer metatarsal IV that is present in a few titanosaurs (e.g. *Alamosaurus*, *Epachthosaurus sciuttoi* and *Notocolossus gonzalezparejasi*) [61]. As in most eusauropods [29], the metatarsal III to tibial length ratio is less than 0.25 in NHMUK PV R16500 (table 1). This contrasts with the higher ratio that characterizes taxa such as *Limaysaurus tessonei*, *Garumbatitan*, *Vouivria*, and especially *Tastavinsaurus*, in which this ratio is 0.3 [36–38]. The proximodistal length of metatarsal III is slightly more than 1.3 times that of metatarsal I (table 1), which is similar to that of most titanosauriforms, contrasting with a lower ratio in most other eusauropods [61]. Metatarsal III is considerably more slender (especially in the main shaft) than metatarsals I and II: this is the apomorphic state that characterizes eusauropod pedes [29].

The proximal articular surface of metatarsal III of NHMUK PV R16500 is mildly rugose, flat dorsoventrally and slightly convex transversely: it therefore lacks the domed surface that characterizes some macronarians [46]. In outline, the proximal articular surface is subrectangular, with concave lateral and medial margins. The long axis of this rectangle is oriented dorsally and moderately laterally such that it is twisted through an angle of approximately 70° with respect to the long axis across the distal end. Thus, the medial and lateral margins of the proximal end face slightly dorsally and ventrally, respectively. The concavity of the lateral margin of the proximal end is more marked than the medial one, largely because the dorsal part of the lateral surface projects laterally and overhangs a fossa for articulation with metatarsal IV. There is a similar, but less prominent, process located at the dorsomedial corner of the proximal end. In dorsal view, the lateral process of the proximal end forms a distinct rounded bulge, making the dorsal profile of the element rather asymmetrical. Proximally, the dorsal surface of the shaft is mildly concave transversely. There is no rugosity on the dorsolateral margin near the distal end.

**Figure 6.**
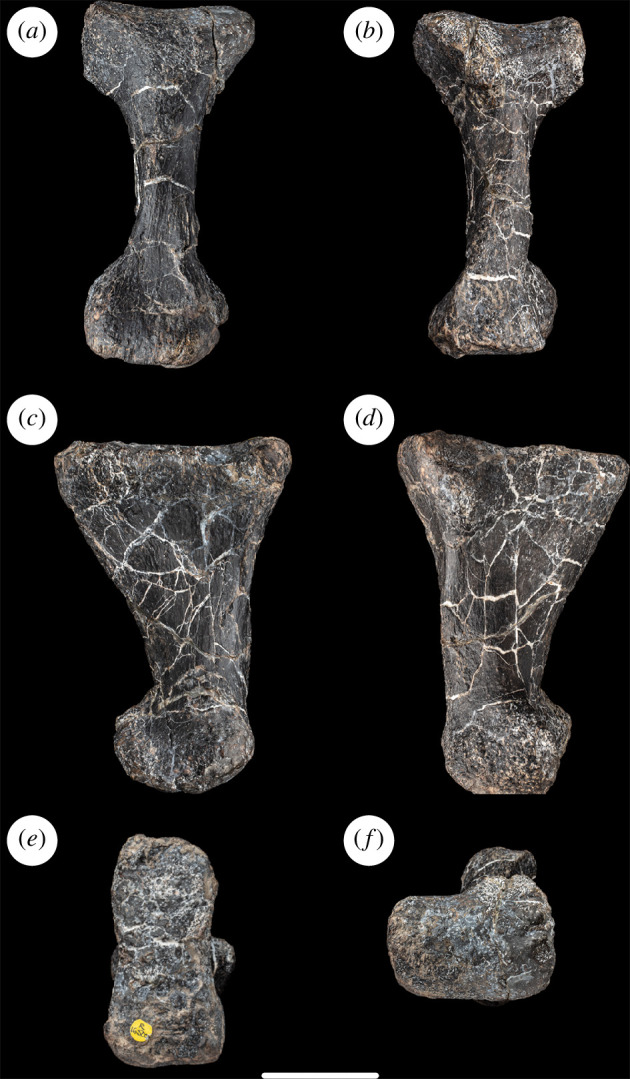
Left metatarsal III of NHMUK PV R16500, in: (*a*) anterior; (*b*) posterior; (*c*) medial; (*d*) lateral; (*e*) proximal; and (*f*) distal views. Scale bar = 50 mm.

The main shaft is strongly compressed transversely such that it is approximately twice as wide dorsoventrally as mediolaterally. The narrow shaft persists until very close to the distal end, at which point the element widens strongly transversely and moderately dorsoventrally to form the distal condylar region. The transverse width of the distal end is approximately 2.9 times that of the main shaft, and the equivalent ratio in metatarsal IV is greater than 3.0. This high distal end to midshaft transverse width ratio is unique among sauropods, as most taxa typically have values close to 2.0 and the next highest ratio observed is 2.3 in *Janenschia* (table 2). The fact that these high ratios occur in both metatarsals III and IV in NHMUK PV R16500, combined with a lack of distortion or other major damage to the shafts, suggests that this feature is a potential autapomorphy rather than a taphonomic artefact (see §5; figure 6).

**Table 2.** Comparison of the ratios of the mediolateral width of the distal end to the minimum mediolateral width of the shaft for metatarsals III and IV in NHMUK PV R16500 and other representative sauropods.

taxon/specimen	metatarsal III	metatarsal IV	source
NHMUK PV R16500	2.9	3.0	this study
*Vulcanodon karibaensis*	1.8	1.7	Cooper [62]
*Tazoudasaurus naimi*	not preserved	1.6	Allain & Aquesbi [63]
*Shunosaurus lii*	1.6	1.5	Zhang *et al.* [64]
*Omeisaurus tianfuensis*	2.1	1.5	He *et al.* [65]
*Turiasaurus riodevensis*	1.9	2.0	P.D. Mannion and P. Upchurch 2009, personal observation
*Janenschia robusta*	2.3	2.2	Mannion *et al.* [14]
*Apatosaurus louisae*	1.8	1.7	Gilmore [66]
*Diplodocus carnegii*	2.2	1.8	Hatcher [67]
*Suuwassea emiliae*	not preserved	1.8	Harris [57]
*Limaysaurus tessonei*	2.0	1.9	P.D. Mannion 2009, personal observation
MAU-Pv-EO−629	1.9	1.8	Bellardini *et al.* [34]
*Camarasaurus lentus*	2.1	1.9	Tschopp *et al.* [35]
‘*Macrurosaurus*’ (CAMSM B55449−55453)	1.9	1.5	P.D. Mannion 2010, personal observation
*Giraffatitan brancai*	2.0	incomplete	Janensch [68]
*Garumbatitan morellensis*	2.1	1.9	Mocho *et al.* [36]
*Gobititan shenzhouensis*	1.9	2.1	P.D. Mannion and P. Upchurch 2007, personal observation
*Tastavinsaurus sanzi* (holotype)	2.2	1.9	P.D. Mannion and P. Upchurch 2009, personal observation
*Ligabuesaurus leanzai*	incomplete	1.9	Bellardini *et al.* [69]
*Diamantinasaurus matildae*	incomplete	1.8	Poropat *et al.* [70]
*Epachthosaurus sciuttoi*	1.6	1.5	P.D. Mannion and P. Upchurch 2013, personal observation
*Alamosaurus sanjuanensis*	1.7	1.5 (slightly incomplete)	D’Emic *et al.* [71]

In dorsal view, the distal articular surface is slightly concave transversely. The distal articulation is convex dorsoventrally and extends a short way onto the dorsal surface and somewhat more strongly onto the ventral surface of the shaft. The medial surface of the distomedial condyle is nearly flat but has a slightly concave profile because of a small medial projection of its ventral margin. The distolateral condyle lacks an equivalent projection and instead has a mildly convex lateral surface. The distomedial condyle is wider dorsoventrally than the lateral one.

#### 4.3.4. Left metatarsal IV

This element is very similar to metatarsal III but is slightly smaller and more gracile. The dorsal margin of the proximal articular surface is straight and wider transversely than the ventral one. The lateral margin is rounded transversely and curves slightly ventromedially. Thus, the lateral margin of the proximal end is slightly convex, whereas the medial one is moderately concave. Similarly, the fossa on the proximal part of the medial surface of the shaft is relatively prominent, whereas on the lateral surface such a fossa is virtually absent. Thus, NHMUK PV R16500 possesses the apomorphic state in which a lateral projection from the upper part of the proximal end of metatarsal III articulates with a medial fossa on metatarsal IV. This condition has previously been noted only in titanosauriforms, including *Alamosaurus*, *Cedarosaurus weiskopfae*, *Garumbatitan*, *Ligabuesaurus*, *Tastavinsaurus* and *Venenosaurus dicrocei* [16,36,71].

**Figure 7.**
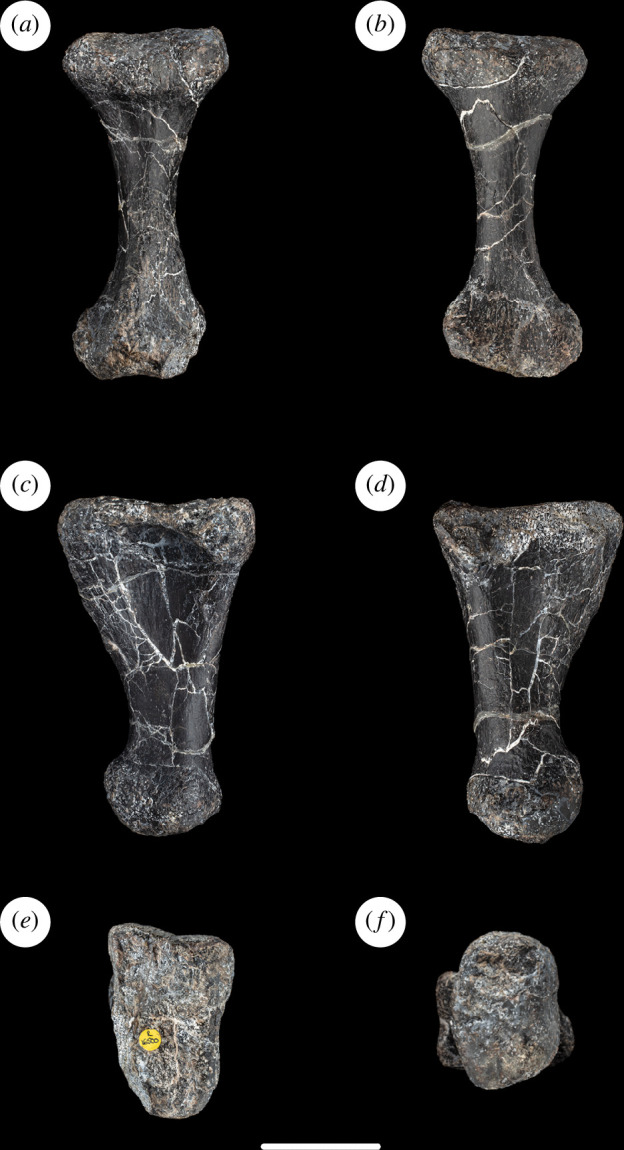
Left metatarsal IV of NHMUK PV R16500, in: (*a*) anterior; (*b*) posterior; (*c*) medial; (*d*) lateral; (*e*) proximal; and (*f*) distal views. Scale bar = 50 mm.

The distal end is wider transversely than tall dorsoventrally. In dorsal view, the distal end surface is approximately perpendicular to the proximodistal axis of the element and thus lacks the apomorphic bevelling that results in it facing somewhat medially in brachiosaurids, *Epachthosaurus* and *Suuwassea* [14,46]. The distomedial condyle lacks the ventral shelf seen in metatarsal III, but its medial surface is still mildly concave and faces slightly proximally. By contrast, the lateral surface of the distolateral condyle is again mildly convex, as in metatarsal III, and thus curves to face slightly dorsolaterally and ventrolaterally towards its margins. Unlike metatarsal III, the distomedial condyle is not taller dorsoventrally than the distolateral one (figure 7).

#### 4.3.5. Left metatarsal V

This element is nearly complete, although it has probably lost the tip of the process projecting from the lateral corner of the proximal end. Metatarsal V is the second shortest of the five metatarsals, only slightly exceeding the length of metatarsal I (table 1). The metatarsals V–IV length ratio is 0.83: values above 0.7 represent an apomorphic enlargement of metatarsal V that characterizes Eusauropoda or a slightly more inclusive clade [39]. In proximal end view, the articular surface is subtriangular in outline, with a long straight ventral margin, and shorter dorsolateral and dorsomedial margins. The two shorter proximal margins meet each other at a domed dorsal apex. This apex lies closer to the proximal medial projection than to the lateral one, such that the dorsolateral margin is longer and slopes more shallowly, whereas the dorsomedial margin is shorter and steeper. In dorsal view, the proximal surface is strongly convex transversely, and it curves distally towards the dorsal apex. The presence of this domed dorsal apex is plesiomorphic for eusauropods, with this morphology retained in taxa including *Dongbeititan*, *Ligabuesaurus* and *Sibirotitan* [44,58,69], but in *Limaysaurus*, *Garumbatitan*, *Tastavinsaurus* and several titanosaurs (e.g. *Alamosaurus*, *Muyelensaurus pecheni*, *Saltasaurus*), the proximal end of metatarsal V has a more dorsoventrally compressed profile and a more uniform thickness along its length [14,36,72].

In NHMUK PV R16500, the subtriangular profile of the proximal end is maintained in the transverse cross-section throughout much of the shaft, with the dorsal apex leading into a low, rounded ridge that extends along the dorsal surface towards the medial edge of the distal end. The junction between the dorsal and ventral surfaces of the main shaft is transversely rounded on the lateral side and slightly more acute on the medial side, with the latter forming a ridge that projects slightly ventromedially and contributes to making the ventral surface of the shaft mildly concave transversely. The ventral surface lacks the tubercle at midlength that occurs in some titanosaurs (e.g. *Epachthosaurus*, *Mendozasaurus neguyelap* and *Saltasaurus* [72]).

**Figure 8.**
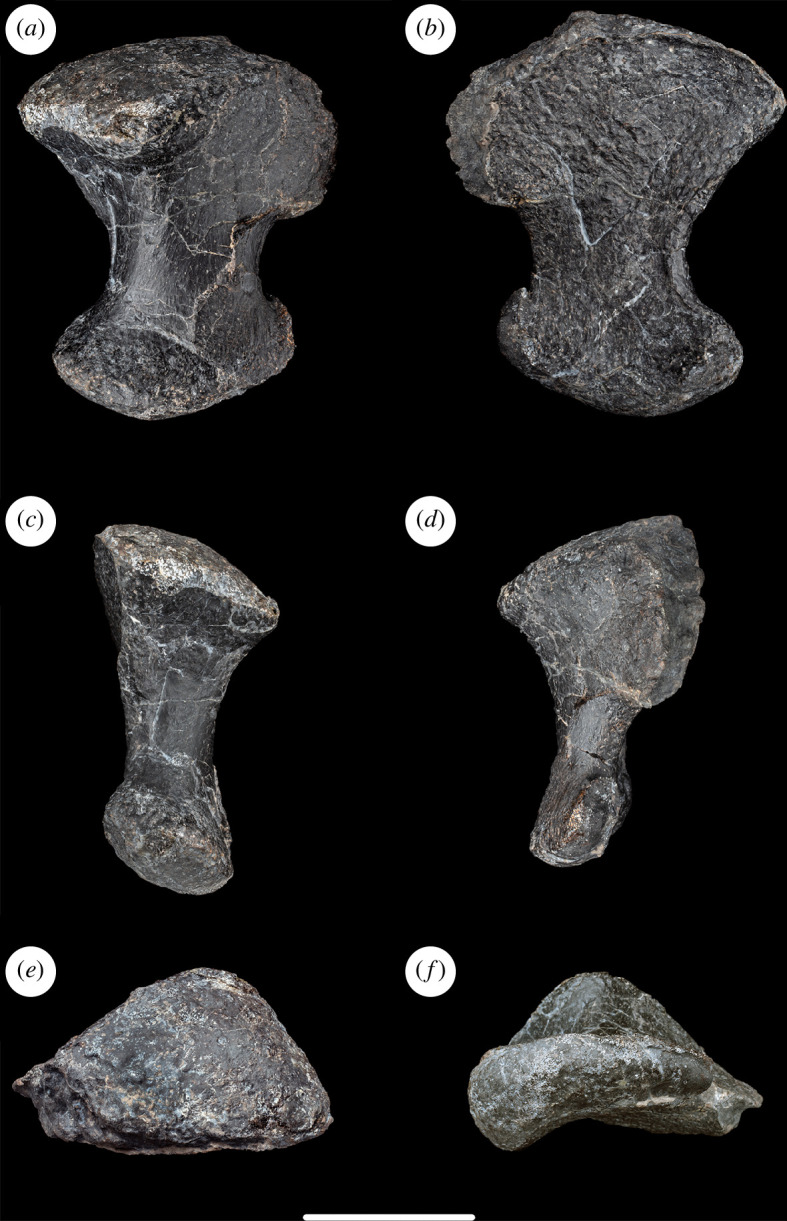
Left metatarsal V of NHMUK PV R16500, in: (*a*) anterior; (*b*) posterior; (*c*) medial; (*d*) lateral; (*e*) proximal; and (*f*) distal views. Scale bar = 50 mm.

With the long axis of the proximal end oriented horizontally, the long axis of the distal end is slightly twisted such that the ventral surface also faces moderately laterally. The distal end is compressed dorsoventrally and widened transversely, though it is not as wide as the proximal end. Nevertheless, the distal end is more strongly expanded than in many other sauropods. This is reflected in a proximal end to distal end transverse width ratio of 1.38: values below 1.6 represent the apomorphic condition that also occurs in taxa such as *Mierasaurus*, *Diplodocus carnegii*, *Cedarosaurus* and *Dongbeititan*, as well as several titanosaurs, including *Malawisaurus* and *Mendozasaurus* [16,35,71,73]. In this regard, metatarsal V of NHMUK PV R16500 differs notably from that of *Tastavinsaurus*, in which this ratio exceeds 2.0 [37]. The distal surface of metatarsal V is convex transversely and dorsoventrally in NHMUK PV R16500, and it is slightly taller dorsoventrally at its medial margin compared with the lateral one (figure 8).

#### 4.3.6. Left phalanx I-1

This element is still attached to metatarsal I, as described above. Its margins are damaged, and one surface is obscured by metatarsal I. As a result, only a few useful anatomical observations can be made. The phalanx is compact, with subequal dorsoventral and mediolateral dimensions that exceed the proximodistal length by approximately 50% (table 1). From what is visible, the proximal and ventral surfaces of phalanx I-1 meet at approximately 90°, as in many eusauropods, but unlike some flagellicaudatans (e.g. *Diplodocus carnegii* and *Suuwassea*) in which this margin is drawn out into a flange that underlies the distal end of metatarsal I [74]. The distal end of phalanx I-1 is convex and nearly semicircular in outline, but with a concave indentation on the ventral margin (figure 4).

#### 4.3.7. Left phalanx II-1

**Figure 9.**
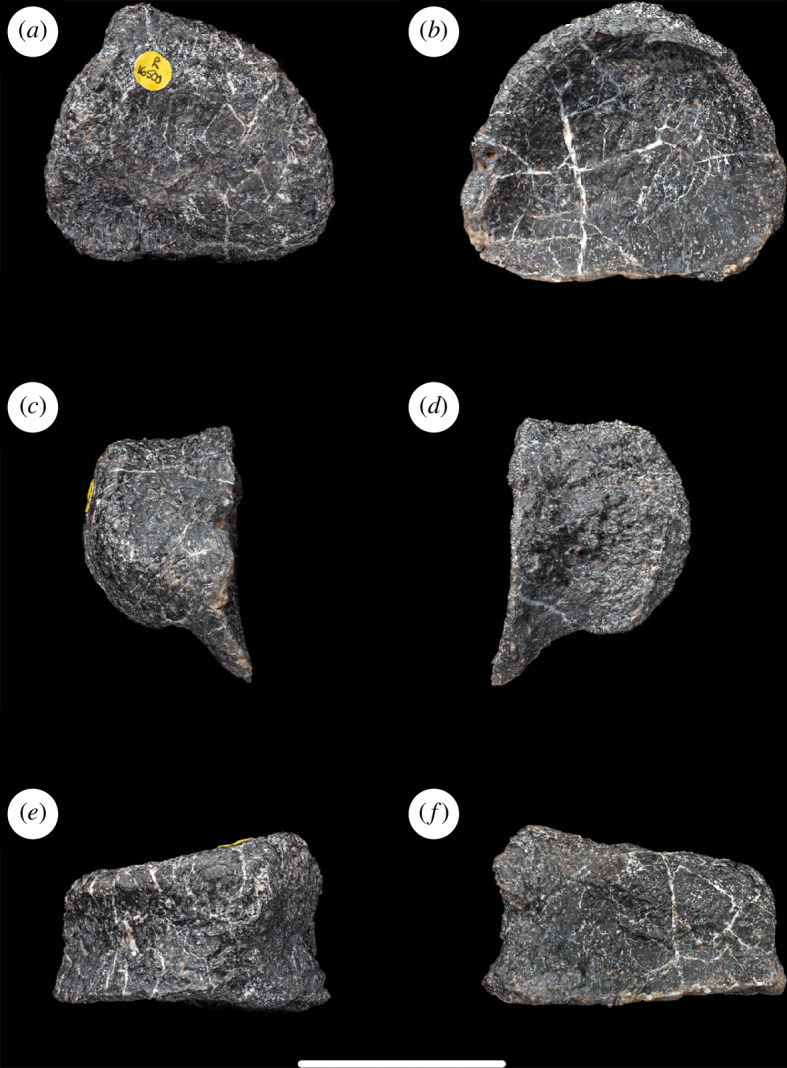
Left phalanx II-1 of NHMUK PV R16500, in: (*a*) anterior; (*b*) posterior; (*c*) medial; (*d*) lateral; (*e*) proximal; and (*f*) distal views. Scale bar = 50 mm.

Phalanx II-1 is the largest non-terminal phalanx. It is wider transversely and dorsoventrally than long proximodistally. The proximal end is ‘D’-shaped in outline, with straight ventral and strongly convex dorsal margins. The proximal articular surface is moderately concave, with its depth increased by the projection of a thin flange from the proximoventral margin. This surface is very subtly divided into two separate areas by a low-rounded vertical ridge, with the more medial fossa being deeper but narrower transversely. The shaft is very short proximodistally and is not strongly constricted relative to the proximal and distal ends. It has a flat medial surface that is steeply inclined and faces slightly dorsally. This meets the dorsal surface of the shaft at an abrupt junction. The dorsal surface of the shaft is convex transversely and curves smoothly into the lateral surface. Thus, the element is tallest dorsoventrally along its medial edge. The ventral surface is flat transversely and mildly concave proximodistally because of the proximoventral flange described above. The distal articular surface is also ‘D’-shaped in outline, and nearly flat transversely because it is not divided into distinct distolateral and distomedial condyles. It is mildly convex dorsoventrally, facing slightly distoventrally and curving onto the ventral surface (figure 9).

#### 4.3.8. Left phalanx III-1

**Figure 10.**
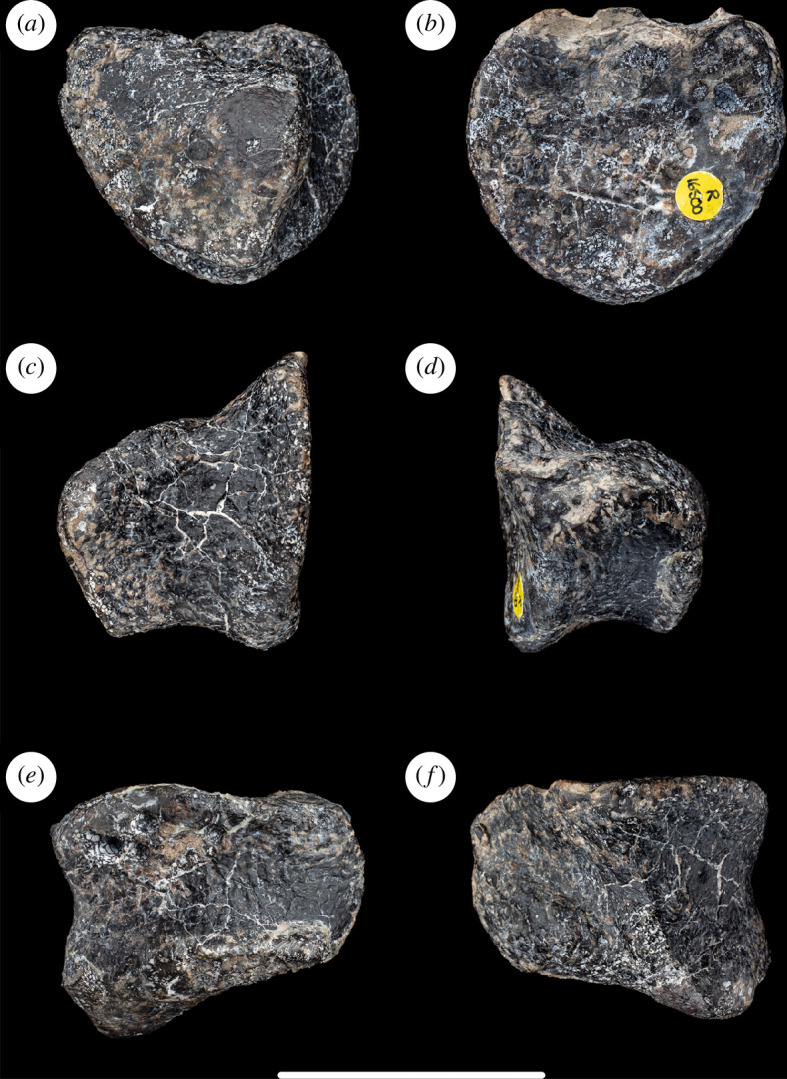
Left phalanx III-1 of NHMUK PV R16500, in: (*a*) anterior; (*b*) posterior; (*c*) medial; (*d*) lateral; (*e*) proximal; and (*f*) distal views. Scale bar = 50 mm.

Phalanx III-1 is intermediate in size and morphology between phalanges II-1 and IV-1, although it resembles the former more closely in most respects. The element is wider transversely and dorsoventrally than long proximodistally, although the latter dimension is longer in relative terms than in phalanges I-1 and II-1 (table 1). The ‘D’-shaped proximal and distal ends resemble those of phalanx II-1, although there is no proximoventral flange. The medial surface of the shaft faces slightly ventrally (figure 10).

#### 4.3.9. Left phalanx IV-1

**Figure 11.**
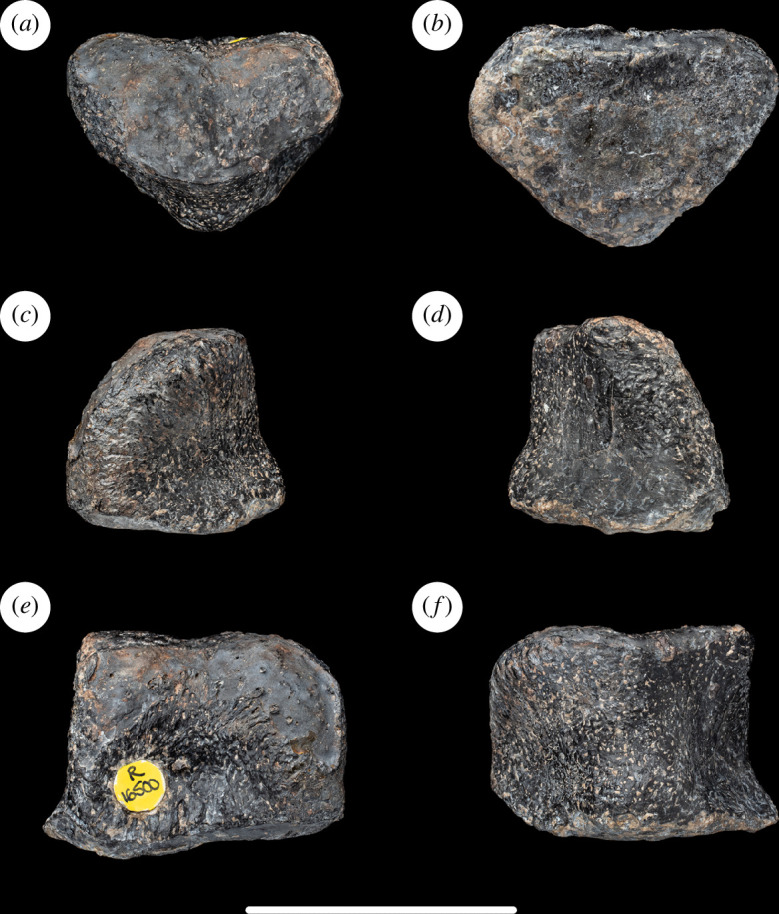
Left phalanx IV-1 of NHMUK PV R16500, in: (*a*) anterior; (*b*) posterior; (*c*) medial; (*d*) lateral; (*e*),proximal; and (*f*) distal views. Scale bar = 50 mm.

Phalanx IV-1 is the smallest of the four preserved non-terminal phalanges. As in the other phalanges, phalanx IV-1 is wider transversely than long proximodistally (table 1). The proximal articular end is very mildly concave and has a rounded subtriangular outline. The main shaft also has a subtriangular transverse cross-section, formed by ventral, dorsomedial and dorsolateral surfaces. The dorsomedial surface is nearly flat, whereas the dorsolateral one is more convex transversely. There is a low midline ridge resulting from where these two surfaces meet dorsally, resembling the unusual condition described in the element interpreted as phalanx II-1 of *Aragosaurus ischiaticus* [75]. The distal end is strongly convex dorsoventrally. It lacks distinct distolateral and distomedial condyles, although it is slightly concave transversely because of subtle medial and lateral ridges. The distal articular surface curves strongly onto the ventral surface of the main shaft but meets the dorsal surface more abruptly (figure 11).

#### 4.3.10. Left ungual phalanges

The ungual phalanges of pedal digits I and II are joined by matrix. They resemble each other except in terms of size and are therefore described together. The two unguals lie side-by-side, with the smaller one (phalanx II-3) projecting further distally than the larger one (phalanx I-2). The latter is damaged and incomplete distally. Nevertheless, the proximodistal length ratio for the ungual of digit I to that of II is greater than 1.34, which indicates that NHMUK PV R16500 possesses the apomorphic increase in proportional difference seen in other gravisaurians (but apparently reversed in titanosaurs [39]). Similarly, the ungual of digit I is longer than metatarsal I (table 1), as is typical for nearly all sauropods [39].

Both the preserved ungual phalanges are transversely compressed such that their proximal articular surfaces are taller dorsoventrally than transversely: thus, NHMUK PV R16500 possesses the apomorphically transversely compressed phalanx II-3 ungual seen in most sauropods, apart from vulcanodontids where the unguals are dorsoventrally compressed [39,63]. The proximal articular surface is mildly concave dorsoventrally and transversely in NHMUK PV R16500. This is a single articular surface: that is, it lacks the plesiomorphic division into medial and lateral articular surfaces separated by a vertical ridge that occurs in non-sauropods and some early-diverging taxa such as *Barapasaurus tagorei* (P. Upchuch 2023, personal observation). In NHMUK PV R16500, a small protuberance is located close to the centre of each proximal articular surface, a potentially unusual condition among sauropods. The proximal surface is bevelled to face proximolaterally so that, when combined with slight lateral deflection of the long axis of the ungual distally, would result in the unguals curving anterolaterally in an articulated pes, as occurs in other eusauropods [29,32,72].

**Figure 12.**
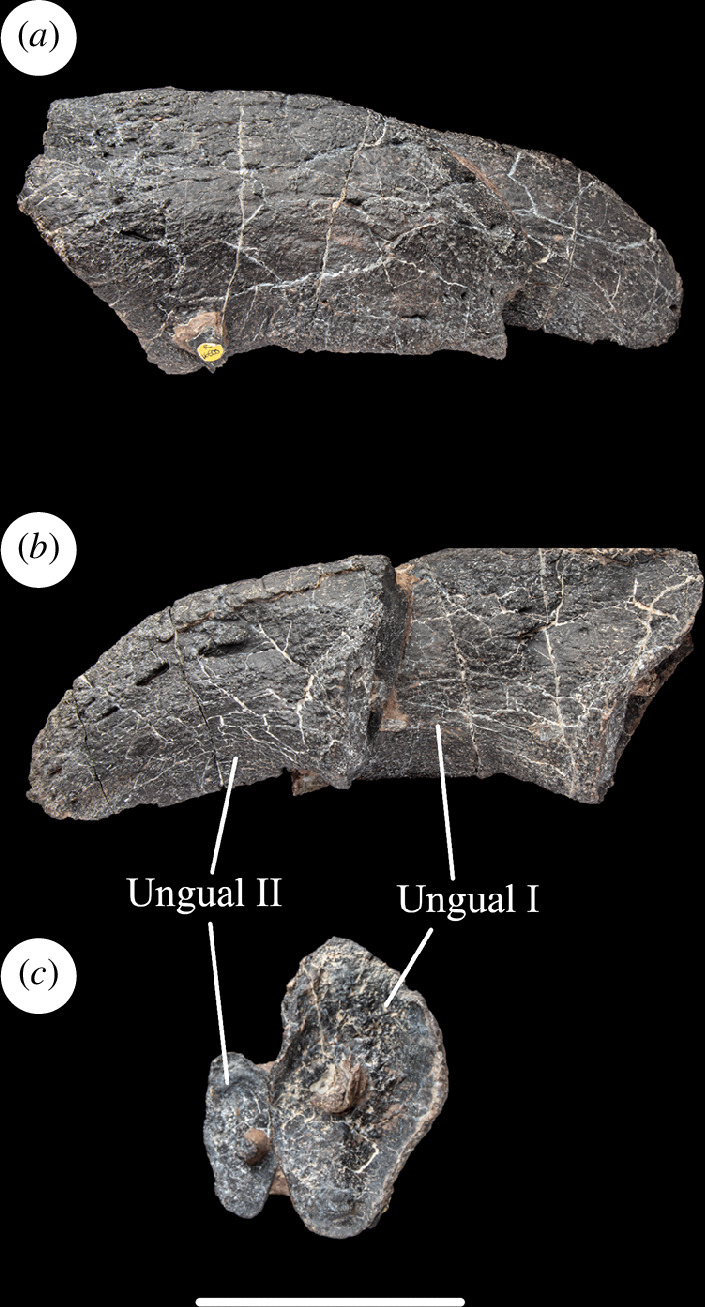
Left phalanges I-2 and II-3 of NHMUK PV R16500, in: (*a*) dorsal; (*b*) ventral; and (*c*) proximal views. Scale bar = 100 mm.

In lateral view, the dorsal and ventral margins are curved and converge towards the blunt distal tip: this creates the typical dorsoventrally wide and recurved claw seen in most eusauropod pedes [29]. The tuberosity on the ventral margin along the distal half of each ungual that characterizes many titanosauriforms, including *Cedarosaurus*, *Garumbatitan*, *Gobititan shenzhouensis*, *Ligabuesaurus* and *Tastavinsaurus* [16,36,37,69], is absent in NHMUK PV R16500, although it is possible that this reflects poor preservation rather than genuine absence. (figure 12).

## 5. Phylogenetic analysis

### 5.1. Phylogenetic dataset and approach

To evaluate the phylogenetic position of NHMUK PV R16500, it was added to the data matrix presented by Poropat *et al.* [70], which is the most recent iteration of that developed by Mannion *et al.* [14,16]. This dataset is the largest currently available for eusauropod taxa, including numerous representatives of all Late Jurassic and Cretaceous clades that are potentially relevant to the identification of NHMUK PV R16500. We further augmented this data matrix with the inclusion of *Garumbatitan*, which had been scored for this data matrix by Mocho *et al.* [36], although we made a small number of changes to the character scores from that study (C270: 1→?; C488: 0→?; C489: ?→0; C490: 0→?). With the addition of NHMUK PV R16500 and *Garumbatitan*, the revised data matrix comprises 128 operational taxonomic units scored for 556 characters. The character list is presented in the electronic supplementary material, file S1, and the Mesquite Nexus and TNT versions of the data matrix are provided in the electronic supplementary material, files S2 and S3, respectively.

The data matrix was analysed in TNT v. 1.6 [76,77], following the protocol used in recent iterations of the dataset [14,70]. The following multistate characters were treated as ordered: 11, 14, 15, 27, 40, 51, 104, 122, 147, 148, 195, 205, 259, 297, 426, 435, 472 and 510. Maximum parsimony analysis of the data matrix was carried out under both equal weights (EQW) and extended implied weights (EIW), with the latter using a *k*-value of 9 and for which we also applied the option to ‘downweight characters with missing entries faster’. We followed Poropat *et al.* [70] by excluding eight taxa *a priori* owing to their phylogenetic instability (*Astrophocaudia, Australodocus, Brontomerus, Fukuititan, Fusuisaurus, Liubangosaurus, Malarguesaurus* and *Mongolosaurus*), with the ‘Cloverly titanosauriform’ and *Ruyangosaurus* additionally excluded from the EQW analyses. An initial new technology search was conducted with sectorial search, drift and tree fusing options selected, and the consensus stabilized five times. The resulting trees then formed the starting point for a traditional search using tree bisection-reconnection. For each of our EQW and EIW analyses, we ran two versions of the analysis in which character 538 (‘astragalus, fibular articular surface: faces laterally or dorsolaterally (0); faces posterolaterally because its anterior margin projects laterally (1)’) was scored as either a ‘?’ or a ‘1’ (note that the version of the matrix presented in the electronic supplementary material scores this character as a ‘?’).

### 5.2. Phylogenetic results

**Figure 13.**
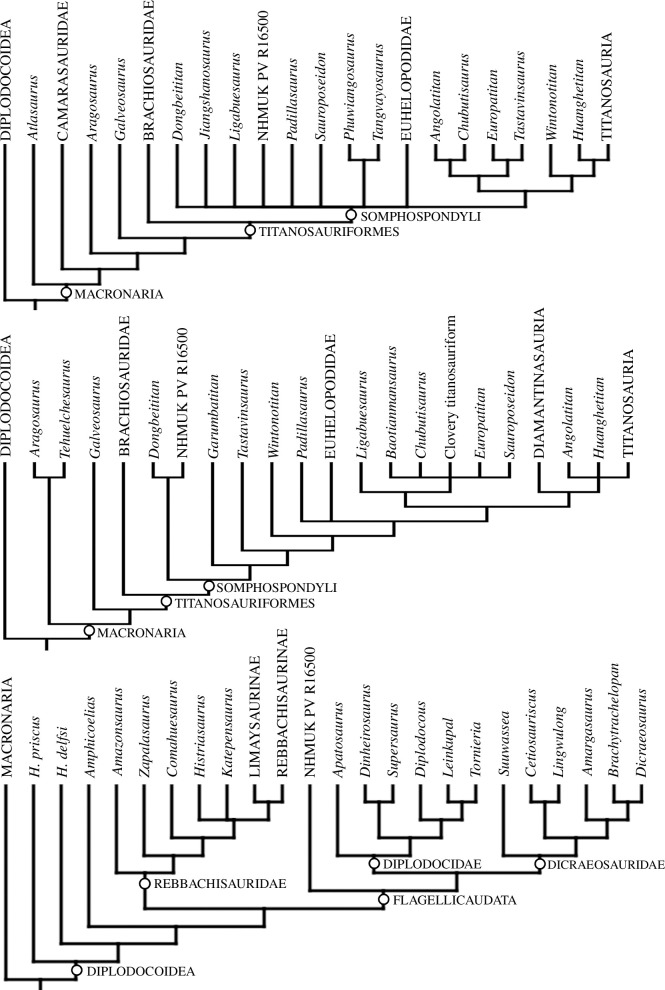
Strict consensus cladograms using (*a*) EQW (with Garumbatitan excluded *a priori*); (*b*) EIW with character 538 scored as a ‘?’; and (*c*) EIW with character 538 scored as a ‘1’. In all cases, only the relevant portion of the neosauropod clade is shown and some lineages are collapsed.

Under EQW with C538 scored as a ‘?’, our analysis yields greater than 9 99 999 most parsimonious trees (MPTs) (i.e. more than TNT can hold) of lengths 2694 steps (Consistency Index (CI) = 0.22, Retention Index (RI) = 0.60). Although parts of the tree are less well-resolved than in previous iterations, and all relevant neosauropod nodes have Bremer supports of 1, the topology is broadly congruent with that of Poropat *et al.* [70]. NHMUK PV R16500 is recovered within a polytomy of early-diverging non-titanosaurian somphospondylans and does not cluster with the European taxa *Europatitan* and *Tastavinsaurus* (figure 13*a*). Both the pruned trees and IterPCR options in TNT identify NHMUK PV R16500 and *Garumbatitan* as among the least stable taxa in the analysis, although these approaches show that NHMUK PV R16500 is a non-titanosaurian somphospondylan that does not cluster with *Garumbatitan*. This placement is also supported in a majority rule consensus tree. We re-ran the analysis with the same settings as before, but with *Garumbatitan* also excluded *a priori*. This reduced the number of steps to 2681 and resulted in slightly greater clarity in the position of NHMUK PV R16500 as a non-titanosaurian somphospondylan. A simplified version of the consensus tree from this analysis is shown in figure 13*a* and the full tree is presented in the electronic supplementary material, file S1. With C538 scored as a ‘1’, there is a one-step increase in tree length, but no other changes to topology or support values for each version of the analysis (i.e. with *Garumbatitan* included and excluded).

Under EIW with C538 scored as a ‘?’, our analysis yields 1 76 400 MPTs with lengths of 115.04 steps (CI = 0.21, RI = 0.59). Aside from a slight loss in resolution, the topology is broadly consistent with that of Poropat *et al.* [70] (see figure 13*b* for the simplified consensus tree, and the electronic supplementary material, file S1 for the complete topology). NHMUK PV R16500 is recovered as the sister taxon of the Early Cretaceous East Asian species *Dongbeititan dongi* (supported by a single synapomorphy pertaining to the morphology of metatarsal V: C74), forming the earliest diverging clade within Somphospondyli. With C538 scored as a ‘1’, there is a slight increase in tree length (115.08 steps), but the number of MPTs and overall topology is unchanged, with the exception of the position of NHMUK PV R16500, which is now recovered as an early-diverging flagellicaudatan, outside of Diplodocidae + Dicraeosauridae (figure 13*c*).

## 6. Discussion

### 6.1. Identification and affinities of NHMUK PV R16500

It is clear from our phylogenetic results that the identification of NHMUK PV R16500 must remain tentative at this stage, and its instability in our tree topologies (figure 13) illustrates the difficulties associated with working on very incomplete fossil specimens [78]. NHMUK PV R16500 can be scored for only 30 out of 556 characters (see below), not only because it preserves just a small part of the original skeleton, but also because of ambiguities caused by postmortem damage and distortion. Moreover, the comparative statements in §4, ‘Description and comparisons’, and the low consistency indices (0.22 and 0.21 for EQW and EIW, respectively), demonstrate that homoplasy is common and widespread in our dataset. Large amounts of missing data and frequent homoplasy both make it difficult to find unequivocal, well-supported phylogenetic relationships. Yet, numerous studies have noted that phylogenetic accuracy tends to increase with better taxon sampling, even when the additional taxa increase the proportion of missing data in the dataset (see review by Tschopp & Upchurch [79].

There is strong support for the placement of NHMUK PV R16500 within Eusauropoda, most probably within Neosauropoda. The specimen possesses numerous synapomorphies of Eusauropoda (or a slightly more inclusive clade), including: (i) the cnemial crest is directed laterally (C260); (ii) tibial distal end transverse to anteroposterior width ratio is less than 1.5 (C68); (iii) the astragalus tapers in anteroposterior and dorsoventral width towards its medial end (this occurs in neosauropods and a subset of eusauropods, C265); (iv) possession of a ridge on the astragalus that separates the posterior surface of the ascending process from the medial fossa (lost in titanosaurs, C552); (v) metatarsal III to tibia proximodistal length ratio is less than 0.25 (C73); and (vi) pedal unguals have bevelled proximal ends so that they are deflected to project anterolaterally (C397). Moreover, eusauropod affinities are supported by characters that are not currently included in the phylogenetic dataset, such as: (i) the tibia has a subcircular proximal end; (ii) reduction of the medial malleolus of the tibia, exposing the posterior surface of the astragalar ascending process in posterior view; (iii) loss of the pit and foramina on the anterior surface of the astragalus, at the base of the ascending process; (iv) the proximal ends of metatarsals I and V are subequal in size to those of metatarsals II–IV; (v) the proximal articular surface of metatarsal I is bevelled relative to the long-axis of the metatarsal, such that the surface faces proximomedially; (vi) the midshaft transverse widths of metatarsals III and IV are reduced to approximately 60% of those of metatarsals I and II; (vii) metatarsal V to metatarsal IV proximodistal length ratio is greater than 0.7; (viii) non-terminal pedal phalanges are wider transversely than long proximodistally; (ix) the ungual on pedal digit I is longer than metatarsal I; (x) the ungual on pedal digit I is more than 1.15 times the length of the ungual on pedal digit II (reversed in titanosaurs); and (xi) pedal unguals are dorsoventrally tall and transversely compressed [29,39,40]. Placement of NHMUK PV R16500 within Neosauropoda is supported by two derived character states: (i) loss of the shelf-like projection from the ventral margin of the astragalar fibular facet (most frequently seen in macronarians, C267); and (ii) the ascending process of the astragalus reaches the posterior margin of the element (this also occurs in some eusauropods closely related to neosauropods, such as *Jobaria,* C266 [16,29,39,40]. Furthermore, we conservatively scored C269 (tongue-like process on the posterior margin of the astragalus) as ‘?’ for NHMUK PV R16500 because the true state might have been affected by poor preservation: however, in the context of a putative somphospondylan affinity for this specimen, the absence of the tongue-like process could be genuine and could reinforce a position within Macronaria [16,46].

Three of our four phylogenetic analyses suggest that NHMUK PV R16500 is an early-branching, non-titanosaurian somphospondylan. Evidence for this relationship is somewhat scanter, but includes: (i) a metatarsal III to metatarsal I length ratio greater than 1.3 (C421); (ii) possession of a process from the proximolateral part of metatarsal III that inserts into a fossa on the proximomedial part of metatarsal IV (C275); and (iii) NHMUK PV R16500 potentially shares a synapomorphic metatarsal V proximal to distal transverse width ratio with the somphospondylan *Dongbeititan* (C74). In short, the identification of NHMUK PV R16500 as a eusauropod, and indeed a neosauropod, seems secure, but its precise position within the latter clade is less well supported, as reflected in our phylogenetic results.

The earliest known putative somphospondylans are represented by *Oceanotitan* from the Kimmeridgian–Tithonian of Portugal ([53]; but see [36] for a non-titanosauriform macronarian position instead) and *Australodocus bohetii* from the Tithonian of Tanzania [14]. Unequivocal somphospondylans are known from the Early Cretaceous, and this clade was probably nearly globally distributed by the latter half of this epoch [36,69,80–82]. The Barremian Isle of Wight sauropod fauna includes: a non-neosauropod eusauropod or possibly early-branching macronarian (*Oplosaurus*); an indeterminate flagellicaudatan or non-neosauropod eusauropod (NHMUK PV R8924 [10,83]); at least one turiasaur [14,22]; one or more rebbachisaurid taxa [7,13,15,84]; one or more non-titanosaurian titanosauriforms that have been variously suggested to represent brachiosaurids or somphospondylans (e.g. *Eucamerotus*, *Ornithopsis* and the undescribed ‘Barnes High’ sauropod [7–9,12,16,20,85,86]; and caudal vertebrae of at least two different titanosaurs [7,19,46,85], one of which has aeolosaurine-like features (see [10] for a review of this fauna). Perhaps the strongest evidence for the presence of early-branching somphospondylans in the Isle of Wight fauna is provided by cervical vertebrae described by Naish *et al.* [20], that share at least two unusual features with *Sauroposeidon* from the Early Cretaceous of North America. Although Naish *et al.* [20] regarded these shared features as indicating brachiosaurid affinities (because *Sauroposeidon* was thought to be a member of that clade at that time), subsequent phylogenetic studies have almost unanimously supported an early-branching somphospondylan placement for this North American taxon [16,46,87]. The identification of NHMUK PV R16500 as an early-branching somphospondylan is thus consistent with our current knowledge of the Isle of Wight fauna and with the wider spatiotemporal range of this clade. However, whether NHMUK PV R16500 is a new taxon or belongs to one of the existing Isle of Wight somphospondylan taxa is uncertain (see §6.2).

A minority result, supported by one of our four phylogenetic analyses, suggests that NHMUK PV R16500 could be an early-branching flagellicaudatan. This is unexpected and would be significant if correct and therefore merits brief discussion. The early-branching position of NHMUK PV R16500, outside of Dicraeosauridae + Diplodocidae, would make this specimen particularly interesting because very few, if any, other taxa have been placed in such a relationship [88]. This would imply a long ghost lineage stretching back at least into the Middle Jurassic [89] and would also provide a glimpse of lower hindlimb morphology just before the dicraeosaurid–diplodocid divergence. Moreover, as noted above, the only specimen previously assigned to a potential flagellicaudatan from the Wessex Formation is a single forked chevron (NHMUK PV R8924 [83]): however, subsequent work has shown that such chevrons also occur in many non-neosauropod eusauropods ([29]; see also [10]). In fact, confirmed Early Cretaceous flagellicaudatans are scarce. These are (i) several dicraeosaurid and diplodocine taxa from the Berriasian–Valanginian Bajada Colorada Formation, Valanginian Mulichinco Formation and Barremian La Amarga Formation of Argentina (e.g. *Amargasaurus* and *Leinkupal* [90–93]); and (ii) indeterminate dicraeosaurid and diplodocine material from the Berriasian–Hauterivian Kirkwood Formation of South Africa [94]. An anterior caudal vertebra from the Aptian–Albian of China [95], a metacarpal from the Valanginian–Barremian of the UK [8], and a trackway at the Berriasian Miraflores I tracksite in Spain [96], have all been interpreted as pertaining to Diplodocidae. However, these Laurasian instances of Early Cretaceous flagellicaudatans are all doubtful. The caudal vertebra was reinterpreted as pertaining to a somphospondylan by Whitlock [78] and probably represents a titanosaur [16]. The metacarpal bears no definitive synapomorphies of Diplodocidae or Flagellicaudata and can only be assigned to Sauropoda [10,95,97]. The diplodocid affinities of the Miraflores trackway have been acknowledged to be ‘tentative’ [96], partly because there are very few preserved manüs of rebbachisaurids and dicraeosaurids available for comparison, and partly because there are few definitive manual or pedal synapomorphies for Diplodocoidea and its subclades that could be manifested in footprints [88].

Thus, if NHMUK PV R16500 could be established as an early-branching flagellicaudatan, it would substantially increase our knowledge of this clade in the Isle of Wight fauna in particular, and the Early Cretaceous of Laurasia more generally. Although all the above factors make a flagellicaudatan identification of NHMUK PV R16500 noteworthy and potentially more significant, they also mitigate against such an unexpected result. Aside from potentially one character, no clear diplodocoid synapomorphies [14,35,47] can be identified in NHMUK PV R16500. The fact that the support for the flagellicaudatan identification is very tenuous at this stage (i.e. largely dependent on whether the fibular facet of the astragalus is considered to face laterally or posterolaterally (C538), and occurs only when homoplasy is penalized under EIW), combined with the stratigraphic incongruence of this potential relationship and the lack of other compelling evidence for flagellicaudatans in the Early Cretaceous of Laurasia, all suggest that this is far less likely than the more conventional and better supported somphospondylan interpretation.

### 6.2. Is NHMUK PV R16500 a new somphospondylan taxon?

A case can be made that NHMUK PV R16500 differs from all known sauropods that preserve pedes and could therefore be diagnosed as a new taxon. There are several clear differences between NHMUK PV R16500 and other European Early Cretaceous somphospondylans such as *Garumbatitan* and *Tastavinsaurus* [36,37] (note: *Europatitan* does not preserve any lower hindlimb material [98] that can be compared with NHMUK PV R16500), including: (i) NHMUK PV R16500 and *Tastavinsaurus* differ in the orientation of the cnemial crest; (ii) *Garumbatitan* and *Tastavinsaurus* possess a second cnemial crest that is absent in NHMUK PV R16500; (iii) the astragalus of NHMUK PV R16500 possesses a ridge that divides the posterior and medial fossae, whereas this is absent in *Garumbatitan*; (iv) metatarsal I of NHMUK PV R16500 lacks the bump-like projection at midlength on the medial surface that is present in *Garumbatitan*; (v) the metatarsal III to tibia length ratio is close to 0.3 in *Garumbatitan* and *Tastavinsaurus*, whereas it is less than 0.25 in NHMUK PV R16500; (vi) the proximal end profile of metatarsal V is subtriangular and ‘domed’ dorsally in NHMUK PV R16500, whereas it is dorsoventrally compressed in *Garumbatitan* and *Tastavinsaurus*; and (vii) the proximal to distal transverse width ratio for metatarsal V is 1.38 for NHMUK PV R16500, but close to 2.0 in *Tastavinsaurus* (see §4). Moreover, NHMUK PV R16500 possesses an unusual pedal phalanx IV-1 morphology, in which the dorsolateral and dorsomedial surfaces meet on the dorsal midline to form the apex of a subtriangular transverse cross-section, a characteristic only previously reported in phalanx II-1 of *Aragosaurus* [75]. There is also an unusual bump-like projection located near the centre of the proximal articular surfaces of the pedal unguals of NHMUK PV R16500, which is potentially autapomorphic. Most notable of all is the autapomorphic widening of the distal ends of metatarsals III and IV, compared with transversely very narrow shafts, such that the distal end to shaft transverse width ratio is approximately 3.0 (compared with values of 2.3 or lower in all other sauropods; table 2).

Despite the presence of these potentially diagnostic features, we have adopted a conservative approach and decided not to erect a new name for NHMUK PV R16500. There are several reasons why this is prudent at this time. First, there are other potential early-branching somphospondylans in the Isle of Wight fauna, including named taxa such as *Ornithopsis hulkei*, *Ornithopsis eucamerotus* and *Eucamerotus foxi*. None of these previously recognized potential somphospondylans preserve any lower hindlimb elements, so they cannot be compared with NHMUK PV R16500. The history of sauropod systematics is littered with examples of confused taxonomy and nomenclature (see reviews in the studies by Upchurch *et al.*, Upchurch & Martin, Wilson & Upchurch, and Taylor [10,99–101]). Such complexity and confusion have often resulted from taxonomic practices in which non-overlapping specimens have been referred to the same taxon based on weak evidence (e.g. geographical and/or stratigraphic proximity, overall anatomical similarity rather than autapomorphy), and the naming of new taxa based on highly incomplete material and/or doubtful diagnostic features. As NHMUK PV R16500 demonstrates, the Isle of Wight continues to yield new sauropod material, and it is conceivable that future discoveries will strengthen the basis for comparing current specimens. For example, the undescribed ‘Barnes High’ sauropod, also from the Wessex Formation of the Isle of Wight, preserves postcranial material including presacral and caudal vertebrae, and girdle and limb elements [10]. At present, the ‘Barnes High’ sauropod is privately owned and unavailable for detailed study, but it illustrates the potential for making more rigorous comparisons and ultimately resolving many of the taxonomic and nomenclatural issues that have plagued UK sauropod systematics since the early work of Seeley, Owen, Hulke, Fox and Lydekker in the late nineteenth century.

A second, and related, reason for not naming NHMUK PV R16500 concerns the concept of historical obsolescence developed by Wilson & Upchurch [100]. Historical obsolescence is the idea that a fossil specimen may bear what appear to be autapomorphies at the time of its discovery, but that these autapomorphies tend to transform into more widespread synapomorphies as new discoveries expand our knowledge. The more incomplete the original type specimen, and the fewer autapomorphies it bears when first named, the more prone it is to historical obsolescence and eventual designation as a *nomen dubium*. NHMUK PV R16500 includes only the lower hindlimb, meaning that it can be scored for only 30 of the 556 characters in the Poropat *et al.* [70] dataset: this gives a character completeness metric (*sensu* [102]) of just 5.4%. Moreover, it bears only two potential autapomorphies. Therefore, we judge this specimen to have a relatively high probability of experiencing historical obsolescence if it were to be named now.

## 7. Conclusion

NHMUK PV R16500 is the lower hindlimb of a neosauropod from the Barremian-aged Wessex Formation of the Isle of Wight, UK. Although incomplete preservation inevitably limits the number of character states that can be scored for phylogenetic analysis, our results suggest that this specimen is most probably an early-branching non-titanosaurian somphospondylan. The less well-supported alternative possibility, that NHMUK PV R16500 is an early-branching flagellicaudatan, would be globally significant for our understanding of diplodocoid diversity and biogeography in the Early Cretaceous, if it could be confirmed: however, this result is supported by only one EIW analysis under specific parameters. We therefore remain cautious and suggest that the more conservative somphospondylan interpretation is preferable at present. There are several other specimens that potentially represent somphospondylans in the Isle of Wight sauropod fauna, but the lack of anatomical overlap makes it impossible to compare them with NHMUK PV R16500. The new specimen, however, does possess at least two autapomorphies, and several other unusual features or combinations of character states, suggesting that it will ultimately play an important role in future studies of Early Cretaceous European sauropod systematics, diversity, and biogeography.

## Data Availability

NHMUK PV R 16500 was donated to, accessioned by, and is publicly available for study at the Natural History Museum, London. All data generated by, and used for, this study is available as part of the electronic supplementary material [103].
